# Comparative Study of Physicochemical Properties and Antibacterial Potential of Cyanobacteria *Spirulina platensis*-Derived and Chemically Synthesized Silver Nanoparticles

**DOI:** 10.1021/acsomega.4c01604

**Published:** 2024-06-26

**Authors:** Ani Harutyunyan, Liana Gabrielyan, Anush Aghajanyan, Susanna Gevorgyan, Robin Schubert, Christian Betzel, Wojciech Kujawski, Lilit Gabrielyan

**Affiliations:** † Department of Biochemistry, Microbiology and Biotechnology, Biology Faculty, Yerevan State University, 1 Alex Manoukian Str., Yerevan 0025, Armenia; ‡ Research Institute of Biology, Biology Faculty, Yerevan State University, 1 Alex Manoukian Str., Yerevan 0025, Armenia; § Department of Physical and Colloids Chemistry, Chemistry Faculty, Yerevan State University, 1 Alex Manoukian Str., Yerevan 0025, Armenia; ∥ Chemical Research Center, Laboratory of Physical Chemistry, 1 Alex Manoukian Str., Yerevan 0025, Armenia; ⊥ The Hamburg Centre for Ultrafast Imaging (CUI), University of Hamburg, Luruper Chaussee 149, Hamburg 22761, Germany; # Institute of Biochemistry and Molecular Biology, Laboratory for Structural Biology of Infection and Inflammation, University of Hamburg, c/o DESY, Notkestrasse 85, Build. 22A, Hamburg 22607, Germany; ¶ European X-Ray Free-Electron Laser Facility GmbH, Holzkoppel 4, Schenefeld 22869, Germany; ∇ Faculty of Chemistry, Nicolaus Copernicus University in Toruń, 7 Gagarina Street, Toruń 87-100, Poland

## Abstract

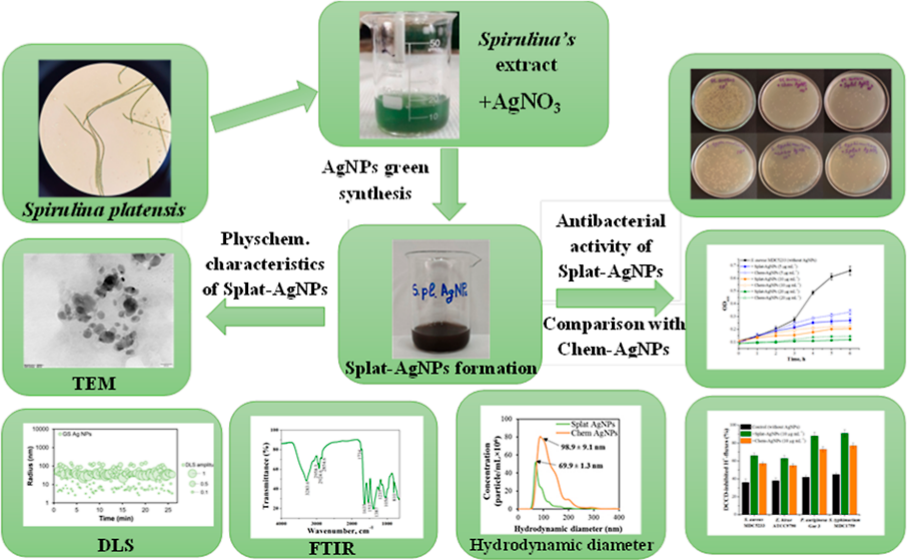

The “green synthesis” of nanoparticles (NPs) offers cost-effective and environmentally friendly advantages over chemical synthesis by utilizing biological sources such as bacteria, algae, fungi, or plants. In this context, cyanobacteria and their components are valuable sources to produce various NPs. The present study describes the comparative analysis of physicochemical and antibacterial properties of chemically synthesized (Chem-AgNPs) and cyanobacteria *Spirulina platensis*-derived silver NPs (Splat-AgNPs). The physicochemical characterization applying complementary dynamic light scattering and transmission electron microscopy revealed that Splat-AgNPs have an average hydrodynamic radius of ∼ 28.70 nm and spherical morphology, whereas Chem-AgNPs are irregular-shaped with an average radius size of ∼ 53.88 nm. The X-ray diffraction pattern of Splat-AgNPs confirms the formation of face-centered cubic crystalline AgNPs by “green synthesis”. Energy-dispersive spectroscopy analysis demonstrated the purity of the Splat-AgNPs. Fourier transform infrared spectroscopy analysis of Splat-AgNPs demonstrated the involvement of some functional groups in the formation of NPs. Additionally, Splat-AgNPs demonstrated high colloidal stability with a zeta-potential value of (−50.0 ± 8.30) mV and a pronounced bactericidal activity against selected Gram-positive (*Enterococcus hirae* and *Staphylococcus aureus*) and Gram-negative (*Pseudomonas aeruginosa* and *Salmonella typhimurium*) bacteria compared with Chem-AgNPs. Furthermore, our studies toward understanding the action mechanism of NPs showed that Splat-AgNPs alter the permeability of bacterial membranes and the energy-dependent H^+^-fluxes via F_o_F_1_-ATPase, thus playing a crucial role in bacterial energetics. The insights gained from this study show that *Spirulina*-derived synthesis is a low-cost, simple approach to producing stable AgNPs for their energy-metabolism-targeted antibacterial applications in biotechnology and biomedicine.

## 1 Introduction

Contemporary medicine has greatly benefited from the discovery, commercialization, and worldwide application of antimicrobial substances, such as antibiotics, for the treatment of different bacterial diseases. However, in recent decades, the rise in resistance to antibiotics in multiple pathogens poses a significant threat to human health.^1,2^ In order to circumvent bacterial resistance to antimicrobial agents, new strategies are required.^3−5^ Nanomaterials are promising substitutes for tackling the challenges of increasing antibiotic resistance.^3,4^ In this context, developing nanoparticles (NPs) with antibacterial properties as prospective new medical agents has become one of the essential areas of research.^5−8^ The prominent feature of employing NPs for microbial growth repression is their distinct influence on various biochemical processes.^6,7^ Furthermore, the growing application of a variety of NPs in healthcare includes implant coatings for bacterial contamination prevention and tissue repair stimulation, and bacteria-sensitive devices for diagnostic purposes and management of harmful bacterial diseases.^6−9^ From this viewpoint, since the early stages of NPs production, silver NPs (AgNPs) have drawn the attention of researchers owing to their high antibacterial and prospective therapeutic potential in comparison with other metal NPs.^6,7,10−13^ AgNPs have a large-surface area, which provides better interaction with microbial cell wall and their penetration through membrane, leading to changes of the membrane permeability and bacterial death.^6,7^ Moreover, the antibacterial effect of AgNPs is more pronounced at low concentrations, in comparison with other NPs, for example iron oxide NPs.^6^

Various NPs are currently produced by applying a range of physicochemical techniques that, despite the benefit of yielding pure particles, are costly and carry numerous environmental dangers. To overcome these threats, eco-friendly and sustainable pathways to synthesize NPs have been implemented. Green technological approaches are favorable since green-synthesized NPs are more robust and nonhazardous than chemically synthesized ones.^13−16^ The utilization of naturally derived biodegradable substances lowers ecological consequences and the cost of technology.^13−16^ Although the biological production of metal NPs is a relatively new research field and till now not been investigated in detail, the “green synthesis” has already enabled the production of stable NPs of various shapes and sizes.^16−18^ Moreover, green synthesized NPs are endowed with various biological activities and have a high potential for application in various fields of biomedicine and biotechnology.^15−18^ Recently, biosynthesis of NPs and their antimicrobial potential have been reported using extracts of Royal Jelly, various plants such as *Stevia rebaudiana*, *Crataegus microphylla*, *Artemisia herba-alba*, *Mentha*, *Rosmarinus*, lichen *Protoparmeliopsis muralis*, and green algae *Desmodesmus abundans* and *Dunaliella salina*.^10−13,19−21^

Microalgae are widely used in biotechnology owing to their ability to produce a large number of bioactive compounds.^22−25^ Several studies reported the biosynthesis of NPs by distinct microalgae and their inhibitory effect on pathogens.^20,21,26−30^ In this way, AgNPs produced by using the extracts of cyanobacteria *Oscillatoria* sp. not only exhibited antibacterial and antibiofilm activity against various pathogens but also demonstrated cytotoxicity against some human breast and colon cancer cell lines.^31,32^ Singh and co-workers reported *Dunaliella*-mediated synthesis of AgNPs with anticancer activity comparable to that of the Cisplatin drug.^21^

*Spirulina (Arthrospira) platensis* is a cyanobacterium that belongs to the phylum Cyanobacteria, Class Cyanophyceae, Order Oscillatoriales, and Family Microcoleaceae. The use of *Spirulina* in biotechnology is a major objective as a useful source of biologically active compounds.^23,25,33^*Spirulina*’s high nutritional value as a natural superfood is owing to its abundance of proteins, fatty acids, vitamins, phycocyanin, and carotenoids.^22,25,33^ Its cells accumulate a large amount (up to 70% of dry weight) of protein, which contain all essential amino acids.^25,33^ Additionally, the antioxidant properties of *Spirulina* make it efficient in prevention of various diseases such as cancer, hyperglycemia, hypercholesterolemia, cardiovascular disease, distinct inflammations, and poisoning from medications and hazardous substances found in the environment.^22,25^ Microalgae are distinctive candidates for the biological synthesis of NPs. There is also an increasing interest in the application of *Spirulina* in NPs production.^30,34,35^ Suganya with co-workers demonstrated that gold NPs biosynthesized using *Spirulina platensis* protein exhibited an antibacterial effect against *Bacillus subtilis* and *Staphylococcus aureus*.^30^ AgNPs synthesized using soluble polysaccharides isolated from *S. platensis* showed significant cytotoxic activity against human hepatocellular carcinoma.^34^ However, until now, there have been a limited number of studies on the green synthesis of AgNPs by *Spirulina* biomass and their biological activity. Moreover, the mechanisms of antibacterial action of *S. platensis*-derived AgNPs have not been explored yet. As the biological production of NPs is a relatively new and understudied area, the biosynthesis of stable AgNPs with antimicrobial properties using the biomass of *S. platensis* will expand the possibilities of their application in various fields of biomedicine and biotechnology.

This study is aimed at presenting a cost-effective and simple method of synthesis that uses biomass of the cyanobacteria *S. platensis* IBCE S-2 to yield stable AgNPs (Splat-AgNPs) and their antibacterial activity against selected conditionally pathogenic Gram-positive (*Enterococcus hirae* ATCC9790, *S. aureus* MDC5233) and Gram-negative bacteria (*Pseudomonas aeuruginosa* Gar 3, *Salmonella typhimurium* MDC1759), which was not reported earlier. The present work is novel to reveal the possible mechanisms of the antibacterial action of Splat-AgNPs via the examination of the energy-dependent H^+^-fluxes across bacterial membranes. Moreover, the first comparative assessment of the antibacterial properties of *S. platensis*-derived silver NPs versus chemically synthesized colloidal AgNPs (Chem-AgNPs) was carried out.

## 2 Materials and Methods

### 2.1 Cultivation Condition of *Spirulina*

**Figure 1:**
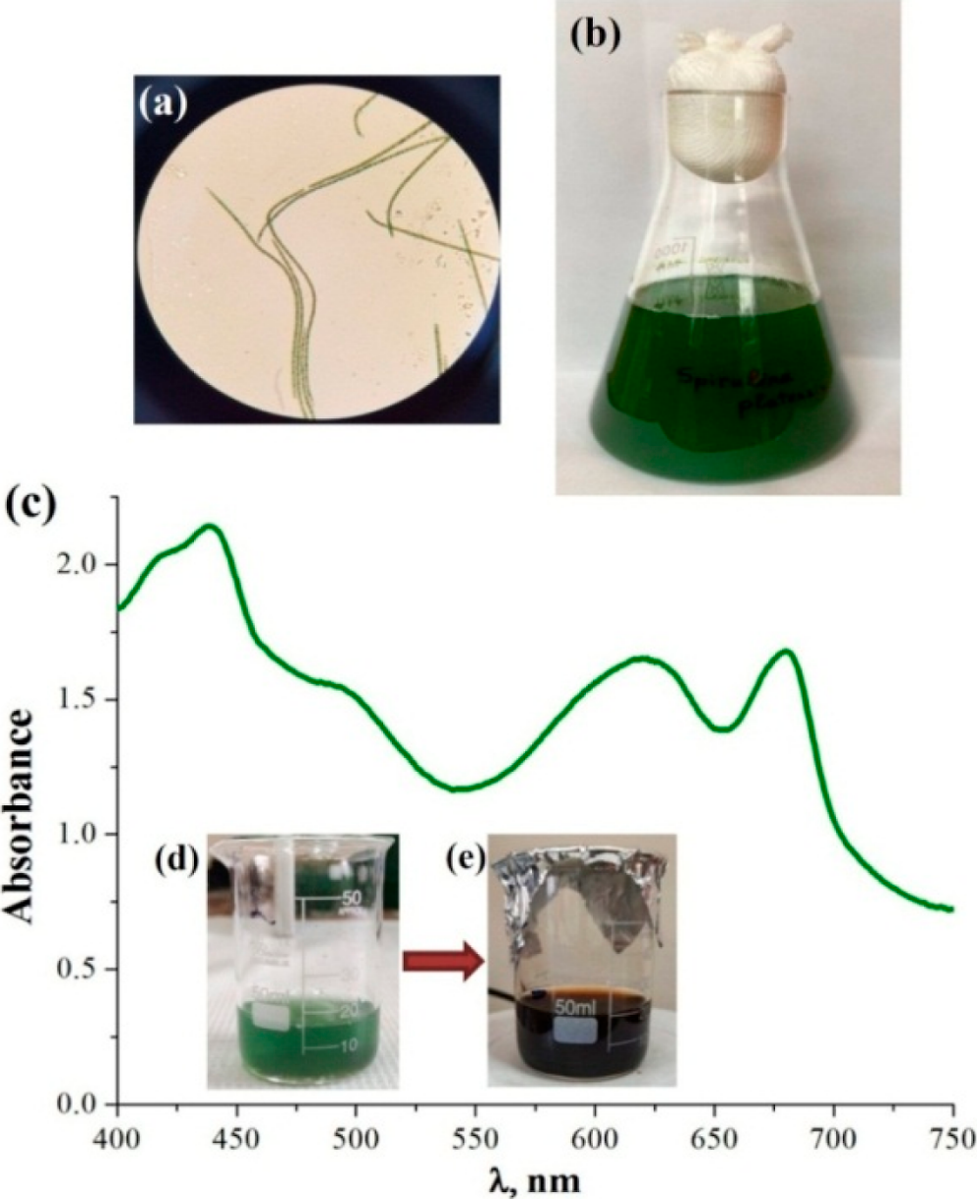
Light microscopy image (×1000, Microscope GT-XSZ-107BN-D, China) (a) and culture of *S. platensis* IBCE S-2 cultivated under aerobic conditions (b); absorption spectrum of *S. platensis* extract (c); and conversion of cyanobacterial biomass (d) to AgNPs (e) by addition of AgNO_3_.

*S. platensis* IBCE S-2 (Algae collection, Institute of Biophysics and Cell Engineering, NAS, Minsk, Belarus) was used for the synthesis of silver NPs (Figure 1a, b). *Spirulina* was cultivated under aerobic conditions in 1000 mL Erlenmeyer flasks containing 500 mL of standard Zarrouk medium [NaHCO_3_ (16.8 g L^–1^), K_2_HPO_4_ (0.5 g L^–1^), NaNO_3_ (2.5 g L^–1^), K_2_SO_4_ (1 g L^–1^), NaCl (1 g L^–1^), EDTA (0.08 g L^–1^), FeSO_4_·7H_2_O (0.01 g L^–1^), MgSO_4_·7H_2_O (0.2 g L^–1^), CaCl_2_·6H_2_O (0.04 g L^–1^), and trace elements solution (1 mL L^–1^)] at 27 ± 2 °C and pH 9.0 ± 0.02 upon a light/dark ratio of 16 L/8 D.^22,36^ The value of optical density at 680 nm was measured for determination of growth of *Spirulina*; and the absorption spectrum of microalga cells was recorded in the wavelength range of 400–750 nm by a Spectro UV–vis Auto spectrophotometer (Genesys 10S UV–VIS-Thermo Fisher Scientific and UV 2700, Shimadzu).^36^

### 2.2 Green Synthesis of AgNPs

*S. platensis* was cultivated under aerobic conditions for 2 weeks (OD_680_ ∼ 2.0); after that cyanobacterial biomass was harvested via centrifugation at 5000 rpm for 15 min (ROTINA 420 R, Hettlich Zentrifugen) and washed twice with water. To obtain *Spirulina*’s aqueous extract, deionized water was added to the precipitate. For the synthesis of Splat AgNPs, 5 mL of *Spirulina*’s extract was added to 45 mL of 1 mM AgNO_3_ solution (1:9 volume ratio) as described.^11^ The reaction mixture (pH 7.0) was shaken at 25 °C for 1 h under 1500 l× illumination. For purification, the synthesized AgNPs were filtered twice using sterile Rotilabo-syringe filters (PVDF, Carl Roth GmbH). The resulting filtrate containing Splat-AgNPs was used for further investigation. In addition, the chemically synthesized AgNPs (Chem-AgNPs; “Silverton”, Armenia) were used for comparative physicochemical characterization and antibacterial potential assessment.

### 2.3 Characterization of AgNPs

Characterization of both Chem- and Splat-AgNPs was performed by using UV–vis spectroscopy. The absorption spectra of pure Chem- and two-fold-diluted Splat-AgNPs were recorded in the range of 280–780 nm, with a 1 nm resolution (Nanodrop 2000C Spectrometer, Thermo Scientific, USA).

Furthermore, in order to reveal the contribution of various functional groups of biomolecules of *S. platensis* in the interaction with Ag^+^, Fourier transform infrared (FTIR) spectroscopy was used.^28,37^ The FTIR spectra of Splat-AgNPs were measured with a resolution of 4 cm^–1^ and 32 parallel scans using a Nicolet iS50 FTIR spectrometer. An attenuated total reflectance technique with ZnSe crystal (incident angle of 45° and 12 reflections) was applied.^38^

Raman spectra of silver NPs were recorded by a Bruker Senterra II Raman microscope using a 532 nm laser wavelength and a 100× objective. Several spots were examined with a focused laser beam to prevent laser-induced damage in the analyzed samples. Before and after each measurement, the samples were inspected by using optical microscopy to ensure that they were not damaged.

The phase composition of the samples was identified by X-ray diffraction (XRD) analysis using a MiniFlex 600 Rigaku SmartLab Standard Error diffractometer (Rigaku Corporation, Japan, D/teX Ultra 250 1D detector, CuKα radiation, λ = 0.1542 nm, step size of 0.02°) and a PDF-2 database. The relative contents of the existing phases were estimated by the Rietveld refinement method.

The elemental composition and purity of Splat-AgNPs were determined by energy-dispersive spectroscopy (EDS) (Prisma E SEM with EDS, ThermoFisher Scientific, USA).

The hydrodynamic dimensions and stability of NPs samples were studied by applying complementary dynamic light scattering (DLS), nanoparticle tracking analysis (NTA), and zeta-potential determination, as described earlier.^10^ For DLS measurements, Splat AgNPs were centrifuged at 1000 g, 20 °C for 30 s (Eppendorf, 5415R, Germany) and then diluted 20-fold in deionized water. Afterward, 90 DLS measurements, each lasting 20 s, were collected (SpectroSize300, XtalConcepts, Germany) for pure Chem- or 20-fold diluted Splat-AgNP solution in a quartz cuvette (Hellma Analytics, Germany). Data and autocorrelation functions were analyzed using a CONTIN algorithm.^39^ Aqueous solutions of 10-fold diluted Chem- and 50-fold diluted Splat-AgNPs were applied for NTA measurements (Nanosight LM10 instrument, Malvern Panalytical, UK). For each sample, five measurements with 60 s duration were recorded, and acquired data were processed using the appropriate software. Mode values are presented.

For zeta-potential determination, either a 10-fold diluted Chem- or 20-fold diluted Splat-AgNP suspension was applied, and five parallel DLS and Phase Analysis Light Scattering measurements were collected (Mobius, Wyatt Technology, USA). The obtained results were analyzed by applying DYNAMICS software (Wyatt Technology, USA), and the averaged values are presented.

Transmission electron microscopy (TEM) and selected area electron diffraction (SAED) were applied for the detailed morphological and crystallinity analysis of Chem- and Splat-AgNP samples. Sample preparation and data collection (JEM-2100-Plus, JEOL, Germany) were performed as described previously.^11,40^

### 2.4 Antibacterial Activity of AgNPs

Selected Gram-negative and Gram-positive bacteria such as *E. hirae* ATCC9790, *P. aeruginosa* Gar3, *S. typhimurium* MDC1759, and *S. aureus* MDC5233 (Microbial Depository Center, NAS, Yerevan, Armenia, WDCM803) were used to reveal the antibacterial potential of Splat- and Chem-AgNPs. The bacterial strains were grown in a nutrient broth (NB) medium at pH 7.5 and temperature 37 °C under anaerobic conditions.^11,41^ Bacteria were cultivated in the presence of Splat-AgNPs and Chem-AgNPs (5, 10, 20, and 30 μg mL^–1^); samples without AgNPs were used as a control. The kinetics of bacterial growth in the presence of AgNPs was monitored by changes in OD_600_ for 6 h; the specific growth rate of bacteria was calculated as described.^11^ The minimal inhibitory concentration (MIC) was determined as the lowest concentration of NPs inhibiting the growth of bacteria.^13^

### 2.5 Bacterial Susceptibility to AgNPs

The bacterial susceptibility to AgNPs was studied by the spread plate method performing the following steps: (i) cultivation of bacteria in the presence of AgNPs; (ii) dilution of the bacterial suspension to 10^8^ colony forming unit (CFU) mL^–1^; (iii) spread of diluted bacterial suspensions (0.1 mL) on agar (1.5%) plates; (iv) incubation at 37 °C; and (v) count of the CFUs number after 24 h.^11,41^

### 2.6 Hemolytic Activity of AgNPs

The hemolytic activity of AgNPs was determined according to the procedure described elsewhere.^41^ The blood was obtained from five healthy donors. Erythrocytes resistance to NPs was measured by the change in the OD_680_ of the erythrocyte suspension by a Spectro UV–vis Auto spectrophotometer (Genesys 10S UV–VIS-Thermo Fisher Scientific, Shimadzu).

### 2.7 H^+^-Fluxes through Bacterial Membranes

The H^+^-fluxes through the bacterial membranes were performed in the following medium: 150 mM Tris-phosphate buffer (pH 7.5), containing 0.4 mM MgSO_4_, 1 mM KCl, 1 mM NaCl, and 0.2% glucose as described elsewhere.^11,41^*N,N′*-dicyclohexylcarbodiimide (DCCD, 0.2 mM), an inhibitor of the H^+^-translocating systems, as well as with Splat- and Chem-AgNPs (10 μg mL^–1^) were added into the assay medium.^11^ DCCD-sensitive H^+^-fluxes were calculated as the difference between the H^+^-fluxes in the presence and absence of DCCD.

### 2.8 Reagents and Statistical Analysis

NB media were purchased from Condalab (Spain); d-glucose (≥98%, anhydrous) and AgNO_3_ (≥99.9%) were purchased from Sigma-Aldrich (USA); Rotilabo-syringe filters (PVDF, 0.22 μm), NaHCO_3_ (≥99%, Ph. Eur., extra pure), K_2_HPO_4_ (≥98%, anhydrous), NaNO_3_ (≥99.0% purity), and other components of Zarrouk medium were obtained from Carl Roth GmbH (Germany). Three independent experiments were performed, based on which the mean ± SD of data measured was calculated. The statistical analysis was carried out by applying Student’s *t*-test, and *p* ≤ 0.05 was considered statistically significant.^11^

## 3 Results and Discussion

### 3.1 “Green Synthesis” of AgNPs Using *Spirulina*’s Biomass

In this study, the *Spirulina*’s biomass (Figure 1b) was applied as a source of reducing and stabilizing agents for the biosynthesis of AgNPs with antibacterial potential. Figure 1c represents an absorption spectrum of *S. platensis* extract with four prominent peaks in the wavelength range from 400 to 750 nm corresponding to the absorbance of chlorophyll a (∼440 and ∼680 nm), carotenoids (∼400–500 nm), and phycocyanin (∼620 nm).

The aqueous extract of *S. platensis*, containing bioactive compounds, leads to the formation of Splat-AgNPs by the bioreduction of Ag^+^ to Ag^0^ (Figure 1d,e). Biosynthesis of Splat-AgNPs was performed under illumination because photon energy is necessary for the AgNPs formation in the presence of “green material”.^21^ Additionally, the reaction was accompanied by agitation to enhance the mass transfer for NPs formation but at the same time avoid particle aggregation. The change in the color of the *Spirulina*’*s* extract from blue-green to dark brown accompanied the biosynthesis of AgNPs, indicating the reduction of Ag^+^ and the formation of NPs (Figure 1d,e). Moreover, the color intensity of the AgNO_3_-containing extract increased after incubation for 24 h. On the contrary, no such change was observed in the extracts not containing AgNO_3_. Therefore, the chosen ratio of the AgNO_3_ solution and *Spirulina* extract was appropriate in terms of AgNP synthesis yield.

### 3.2 Physicochemical Characterization of the Green and Chemically Synthesized AgNPs

**Figure 2:**
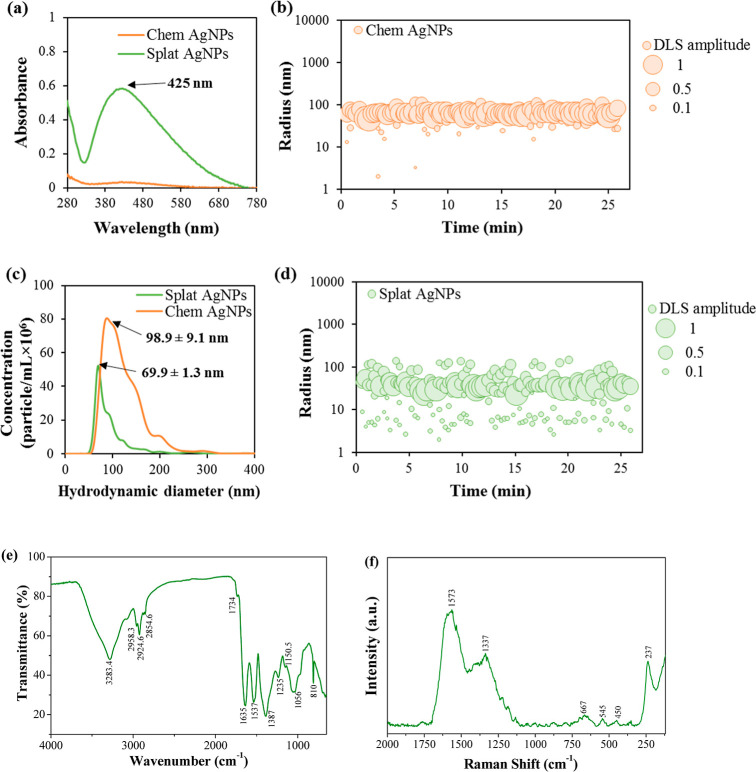
UV–vis spectra (a), hydrodynamic radii (b,d), and correlation of the hydrodynamic diameter with concentrations (c) of Chem- and Splat-AgNPs; FTIR (e) and Raman (f) spectra of Splat-AgNPs.

Green synthesized Splat and Chem-AgNPs were subjected to comparative physicochemical characterization. The surface plasmon-conditioned optical properties of metal NPs enable UV–vis characterization.^42^ Hence, for the Splat-AgNPs, a UV–vis absorption peak at ∼ 425 nm was recorded, whereas Chem-AgNPs displayed no absorbance in the applied wavelength range (Figure 2a). In agreement with earlier reports, the absorbance in the 400–500 nm range confirms the formation of Splat-AgNPs.^43^ However, it is noteworthy that the absorbance maximum and peak broadness are affected by the size and shape of NPs, as well as the solvent molecules.^10,43^ The single blue-shifted absorbance peak of Splat-AgNPs is a primary indication of relatively small and morphologically consistent particles. Subsequent characterization of the hydrodynamic dimensions and morphological examination provided supportive data for this.

The hydrodynamic dimensions of NPs suspensions were investigated on the basis of particle Brownian motion fluctuations and trajectory tracking.^44,45^ According to the DLS and NTA results, both Chem- and Splat-AgNPs have nanoscale dimensions (Figure 2). Chem-AgNPs demonstrated a hydrodynamic diameter of ∼ 98.9 ± 9.1 nm and a hydrodynamic radius of ∼ 53.88 ± 11.65 nm with a polydispersity index (PDI) of 27.3% (Figure 2c, Table 1). Compared to this, Splat-AgNPs revealed a higher polydispersity of 36.5%, containing particles up to ∼ 100 nm in size, but a smaller hydrodynamic radius of ∼ 28.70 ± 5.40 and a hydrodynamic diameter of ∼ 69.9 ± 1.3 nm of the main fraction (Figure 2c, Table 1). In addition, particle concentrations of Chem- and Splat-AgNPs determined by NTA are shown in Table 1.

**Table 1:** Hydrodynamic Radius, PDI, Zeta-Potential, and Concentrations in Suspensions of Chem- and Splat-AgNPs

sample	hydrodynamic radius, nm	polydispersity index, %	zeta-potential, mV	concentration [particle/mL × 10^9^ ± standard error (SE)]
Chem-AgNPs	53.88 ± 11.65	27.3	–52.20 ± 4.10	6.34 ± 7.86 × 10^8^
Splat-AgNPs	28.70 ± 5.40	36.5	–50.00 ± 8.30	1.92 ± 4.94 × 10^7^

Time-resolved DLS measurements during ∼ 30 min revealed no significant changes in the hydrodynamic radii of both Chem- and Splat-AgNPs (Figure 2b,d), which is a sign of particle stability. However, the stability of NPs was confirmed by determining zeta-potential values, which is a well-known approach for colloidal stability assessment.^46^ The obtained results showed that both Chem- and Splat-AgNPs have negative zeta-potential values, (−52.20 ± 4.10) and (−50.0 ± 8.30) mV, respectively. The presence of such high negative values prevents aggregation of particles due to repulsion.^10,47^

The presence of several intense bands at 3283.4, 2958.3, 2924.6, 1635, 1537, 1387, 1235, 1056, and 810 cm^–1^ in the FTIR spectrum of biosynthesized AgNPs characterizes the fundamental vibrational modes of various functional groups of biomolecules of *S. platensis* (Figure 2e). The wide band observed at 3283.4 cm^–1^ is typically assigned to the N–H and O–H stretching vibrations of the secondary amine and hydroxyl functional groups of biomolecules. The FTIR spectrum of Splat-AgNPs shows two sharp peaks at 1635 and 1537 cm^–1^ corresponding to amide I (CO stretching vibration) and amide II (combination of N–H bending vibration and C–N stretching vibration), respectively.^12^ It is worthwhile to note that a peak corresponding to amide I in *S. platensis* extract spectrum was observed at 1644 cm^–1^, i.e., it shifts from a higher wavenumber to a lower (1635 cm^–1^), which suggests the direct participation of C = O group (amide I) in the process of Splat-AgNPs generation (Figure 2e). *S. platensis* extract has a high lipid composition, as evidenced by peaks related to C–H stretching vibrations between 2958 and 2855 cm^–1^, CO stretching vibration of the carboxylic group at 1734 cm^–1^, as well as C–O–C stretching vibration between 1235 and 1056 cm^–1^. The strong band at 1387 cm^–1^ mainly corresponds to antisymmetric N–O stretching in the nitrate groups. The intense band in the range 810–650 cm^–1^ indicates the Ag–O bond formation.^37^

Using Raman spectroscopy, the composition of the surface constituents of AgNPs was revealed. The Raman spectrum of AgNPs is shown in Figure 2f and exhibits bands at 237, 450, 545, 667, 1337, 1573, and 2933 cm^–1^ (the last one is not shown). The presence of broad bands at 1337 and 1573 cm^–1^ is due to the symmetric vibrational modes of various functional groups of biomolecules of *S. platensis*, mainly carboxyl and/or C–N groups, and the weak band at 2933 cm^–1^ is associated with the stretching vibration of the C–H group. The next strong band in the Raman spectrum appears at 237 cm^–1^ and is assigned to the Ag–O symmetric stretching mode.^48^ The weak bands located approximately at 667, 545, and 450 cm^–1^ can be related to carboxyl and C–N group bending as well as Ag–O vibrational modes.

**Figure 3:**
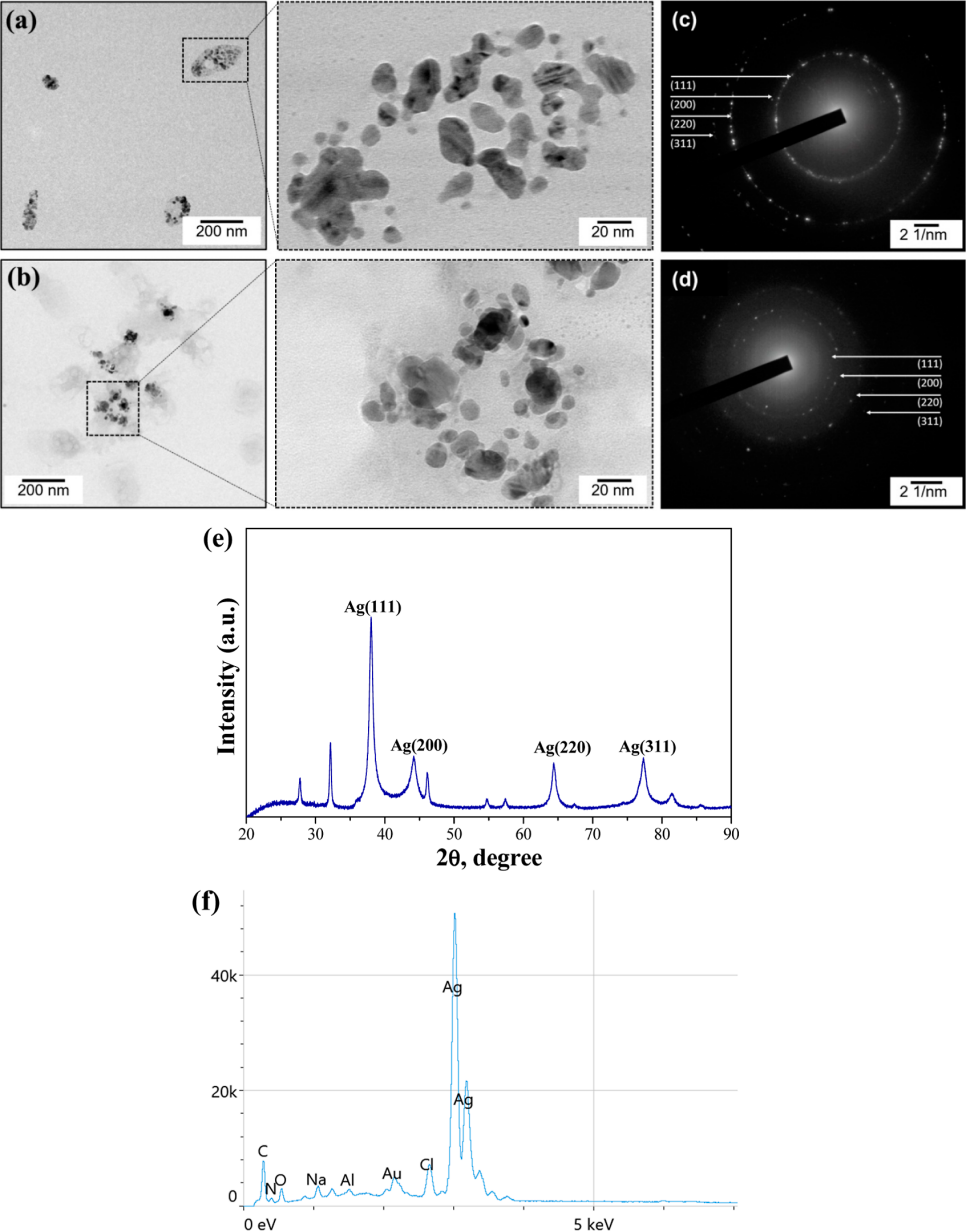
TEM micrographs of Chem-AgNPs (a) and Splat-AgNPs (b); SAED patterns of Chem-AgNPs (c) and Splat-AgNPs (d); XRD pattern of Splat-AgNPs (e); and EDS spectrum of Splat-AgNPs (f).

TEM results (Figure 3a,b) showed that Splat-AgNPs have mainly spherical morphology, whereas Chem-AgNPs can be described as more elongated and irregular-shaped. The size and shape of AgNPs have been reportedly linked to the temperature and pH conditions for the synthesis.^13,49^ Synthesis at acidic pH and lower temperature induces particle aggregation, whereas small and spherical AgNP formation is favorable in the pH range of 7.0 or higher and correlated with a temperature increase.^49,50^ Furthermore, the bioreduction in neutral or alkaline mediums yields highly stable AgNPs.

Additionally, SAED analysis confirmed the crystallinity of both Chem- and Splat-AgNPs (Figure 3c,d), and the Miller indices (111; 200; 220 and 311) match the expected values reported.^51,52^ Moreover, the XRD pattern of Splat-AgNPs (Figure 3e) revealed the presence of intense peaks at 2θ values of 37.96, 44.19, 64.34, and 77.25° corresponding to (111), (200), (220), and (311) reflection. The values of interplanar spacing of these diffraction peaks determined by Bragg’s law were 0.2369, 0.2048, 0.1445, and 0.1234 nm, respectively. The strongest reflection from the (111) diffraction peak indicates a face-centered cubic structure of NPs with a lattice constant of a = 4.0861 Å. The XRD pattern suggests that the biosynthesized NPs are well crystallized.^20,43^

To confirm the purity of biosynthesized AgNPs, EDS analysis was performed. The EDS spectrum of Splat-AgNPs showed typical strong signals approximately at 3 keV, indicating the predominance of Ag content in the sample as well as the purity of Splat-AgNPs (Figure 3f). These results are in the good agreement with the data obtained by other researchers.^13,35^

### 3.3 Comparative Analysis of the Antibacterial and Hemolytic Potential of the Green and Chemically Synthesized AgNPs

The antibacterial potential of Splat- and Chem-AgNPs was evaluated against conditionally pathogenic Gram-positive *S. aureus* and *E. hirae* and Gram-negative *P. aeruginosa* and *S. typhimurium*. Among the representatives of *Enterococcus* and *Salmonella* genera, pathogenic forms are distinguished that cause various human diseases, such as infections of the gastrointestinal tract, genitourinary system, or central nervous system.^53,54^ On the other hand, conditionally pathogenic *S. aureus* and *P. aeruginosa* are associated with nosocomial diseases.^55,57^ All bacteria used demonstrated multidrug resistance against various antibiotics, such as ampicillin, penicillin, cefotaxime, and cefepime.^56^

**Figure 4:**
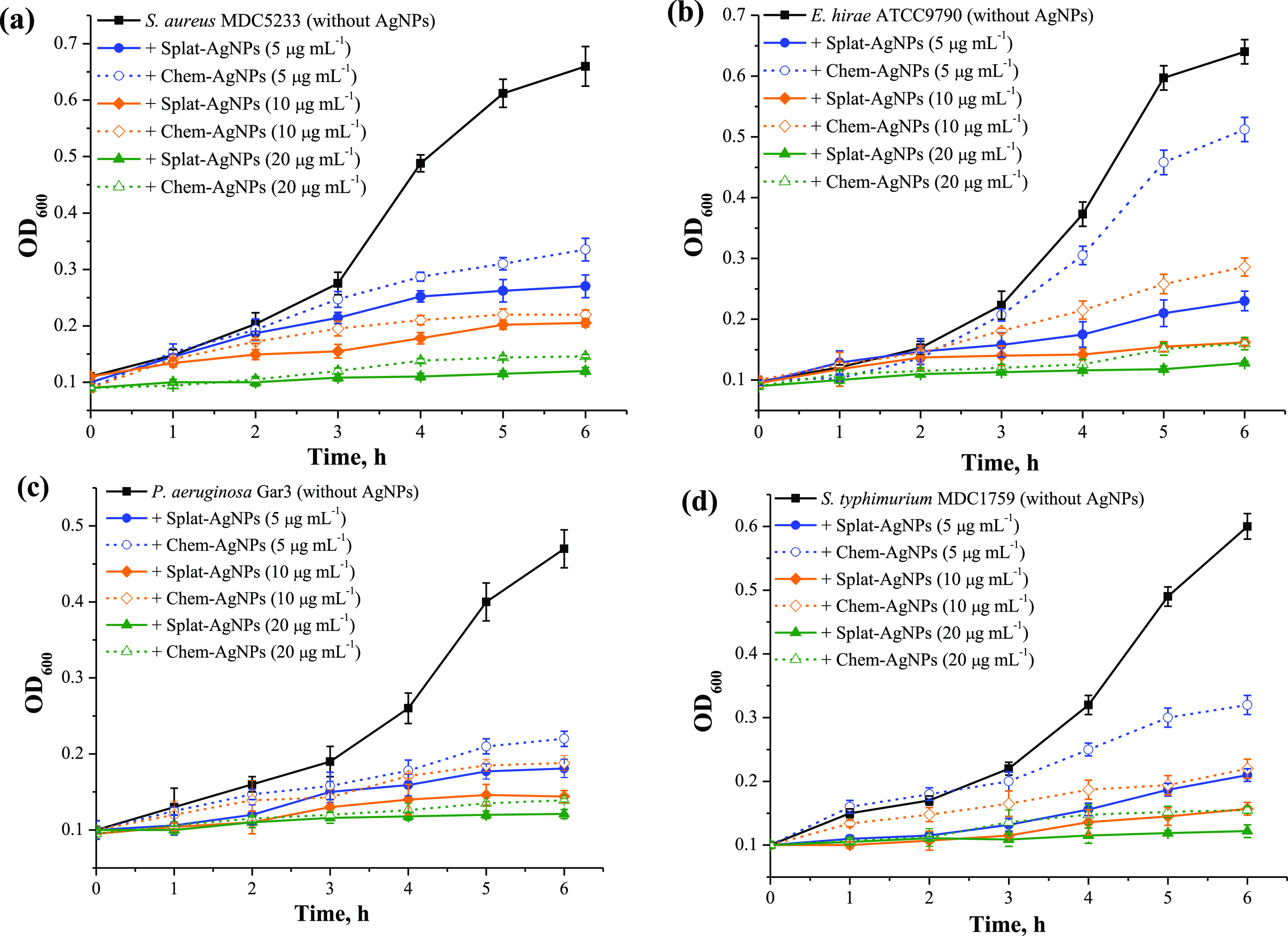
Growth kinetics of Gram-positive bacteria *S. aureus* (a) and *E. hirae* (b) and Gram-negative bacteria *P. aeruginosa* (c) and *S. typhimurium* (d) in the presence of Chem- and Splat-AgNPs. Control bacteria were cultivated without AgNPs.

Figure 4 represents the growth kinetics of Gram-positive and Gram-negative bacteria. Both Splat- and Chem-AgNPs demonstrated antibacterial potential and a concentration-dependent inhibitory effect on the growth kinetics of selected bacterial strains (Figure 4). At concentrations of 5–20 μg mL^–1^, NPs suppressed growth of investigated bacteria during 6 h of cultivation; moreover, Splat-AgNPs exhibited a more pronounced bactericidal effect compared to Chem-AgNPs.

**Figure 5:**
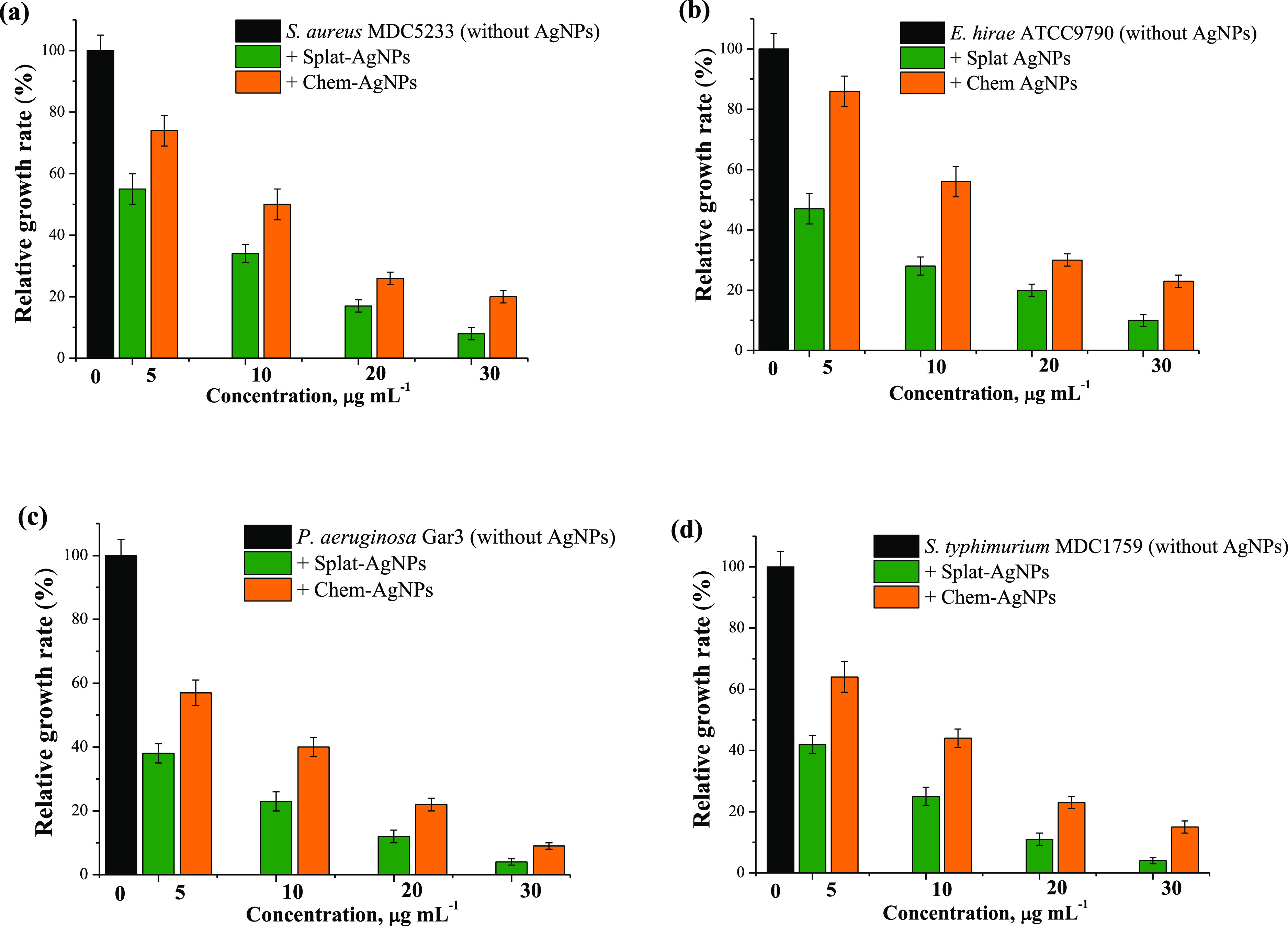
Antibacterial activity of Chem- and Splat-AgNPs against *S. aureus* (a), *E. hirae* (b), *P. aeruginosa* (c), and *S. typhimurium* (d). Control is bacteria cultivated without AgNPs.

The bactericidal activity of Splat- and Chem-AgNPs against Gram-positive *E. hirae* and *S. aureus* and Gram-negative *P. aeruginosa* and *S. typhimurium* estimated by the change in the growth rate of bacteria is shown in Figure 5. Splat-AgNPs demonstrated a more pronounced antibacterial effect on the growth rate of Gram-negative bacteria (Figure 5). In this manner, the addition of 5 μg mL^–1^ Splat-AgNPs decreased the growth rate of *S. aureus* and *E. hirae* by ∼45 and 50%, respectively, whereas in the case of Gram-negative *P. aeruginosa* and *S. typhimurium*, ∼60% decrease was observed (Figure 5).

The difference in AgNPs action on Gram-positive and Gram-negative bacteria is related to the structure of their cell wall, which demonstrates different behaviors for NPs adsorption. Gram-positive bacteria contain a thick peptidoglycan cell wall, which can act as a barrier to AgNPs, while the cell wall of Gram-negative bacteria with a thin peptidoglycan layer and an outer membrane with pores can facilitate penetration of NPs.^6,10,30^ NPs can interact with cell wall proteins, cause changes in membrane permeability, and as a result destroy the bacterial metabolism.^6,12,41^ Razavi with co-workers reported a strong antibacterial effect of AgNPs synthesized using various plants aqueous oil extract against Gram-negative and Gram-positive bacteria due to the increase in membrane permeability and disruption of bacterial cell wall integrity.^12^ AgNPs can inhibit the growth of Gram-negative bacteria *Escherichia coli* owing to formation of pits in the cell wall, leading to increase of membrane permeability and cell death.^6^ The interaction of NPs with bacterial cells is coupled with the charge of NPs, for example, positive charged AgNPs showed a more pronounced antimicrobial effect compared to the negative charged NPs.^6,57^ Additionally, as we have reported previously, iron oxide NPs (with round-shaped morphology and an average size of ∼ 10 nm) also exhibited a more noticeable effect on Gram-negative *E. coli*, compared to Gram-positive *E. hirae*.^41^

The bacteria tested demonstrated less susceptibility to Chem-AgNPs compared with Splat-AgNPs (Figure 5). Moreover, Splat-AgNPs showed the MIC at < 5 μg mL^–1^, whereas MIC of Chem-AgNPs was 10 μg mL^–1^. It should be noted that MIC value of biosynthesized NPs is compatible with MIC values of *Crataegus microphylla* fruit extract-mediated AgNPs reported by Mortazavi-Derazkola et al.^13^ In the presence of 5 μg mL^–1^ Chem-AgNPs, the growth rates of *S. aureus* and *E. hirae* decreased by ∼26 and ∼14%, respectively (Figure 5a,b). At a concentration of 10 μg mL^–1^, Chem-AgNPs suppressed the growth rate of *S. aureus* and *E. hirae* by ∼50 and 46%, correspondingly, whereas Splat-NPs inhibited by ∼66 and 72%, compared to the controls (Figure 5a,b). The same concentration of Chem-AgNPs reduced the growth of Gram-negative *P. aeruginosa* and *S. typhimurium* by ∼60 and 56%, respectively, while in the presence of Splat-AgNPs, a ∼75% decrease was observed for both bacterial strains (Figure 5c,d).

**Figure 6:**
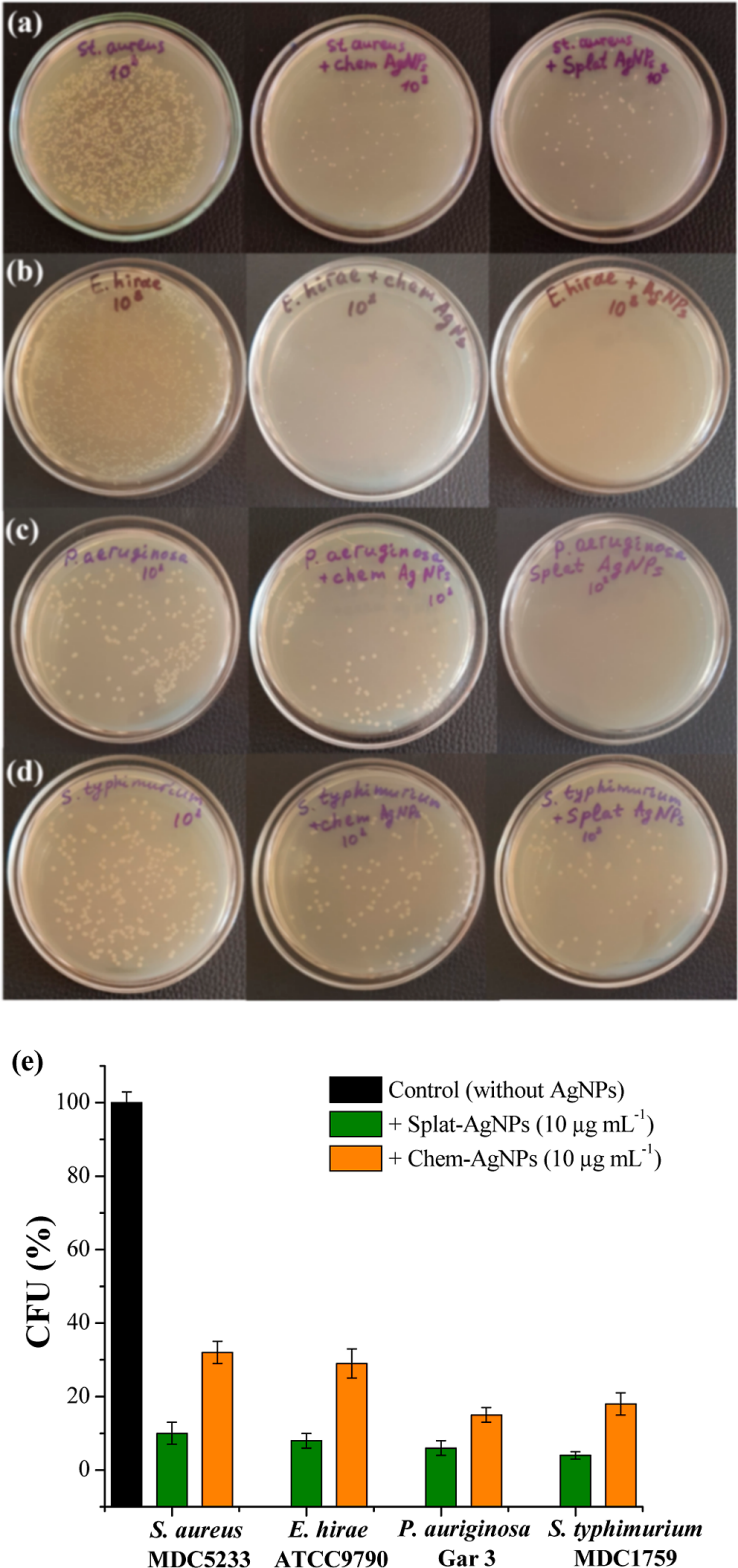
Viable colonies of *S. aureus* (a), *E. hirae* (b), *P. aeruginosa* (c), and *S. typhimurium* (d) cultivated without the addition of NPs and in the presence of Chem- and Splat-AgNPs (left to right). The CFUs of bacteria cultivated in the presence of Chem- and Splat-AgNPs (e). Control is bacteria grown without AgNPs.

Figure 6 represents the effect of 10 μg mL^–1^ Splat- and Chem-AgNPs on CFUs of Gram-positive and Gram-negative bacteria. According to the data obtained, Splat-AgNPs display a noticeable antibacterial effect against bacteria tested in comparison with Chem-AgNPs (Figure 6). The difference between the antibacterial effects of Splat- and Chem-AgNPs is coupled with their size and morphology: Splat-AgNPs have mainly spherical morphology with an average radius size of ∼29 nm, whereas Chem-AgNPs are irregular-shaped with an average radius size of ∼54 nm. Therefore, the antibacterial effect of AgNPs is size-dependent.^10^ Splat-AgNPs have a higher ability to interact with the bacterial membrane and penetrate the cell, thereby inhibiting bacterial growth. Additionally, various bioactive compounds of the *S. platensis* extract surrounding NPs also contribute to the higher antibacterial potential of Splat-AgNPs.^27,29^ Hence, the use of microalgae biomass, especially *Spirulina*’*s*, in the green synthesis of NPs is advantageous. Moreover, Splat-AgNPs demonstrated a pronounced antimicrobial effect in comparison with known antibiotics, such as ampicillin, penicillin, cefotaxime, or cefepime. Thus, the antimicrobial efficacy of AgNPs is mainly affected by the conditions of NP synthesis (pH, temperature, and light intensity) and their characteristics such as shape, size, charge, and composition of surface constituents.^6,13,50^

Currently, AgNPs are widely used in biomedicine, and the cytotoxicity of AgNPs can prevent their application in diagnostics and therapy.^5,7,9^ It was reported that the hemolytic activity of NPs indicates their biocompatibility with blood cells.^58^ Hemolysis is the process of release of hemoglobin from erythrocytes into the plasma, which is caused by damage of the erythrocyte membranes. The hemolytic potentials of Chem- and Splat-AgNPs have been determined to reveal their compatibility with erythrocytes. The results obtained indicate that both AgNPs do not exhibit any hemolytic activity against erythrocytes at the low concentrations tested.

### 3.4 Effects of AgNPs on the Energy-Dependent H^+^-Fluxes through Bacterial Membranes

**Figure 7:**
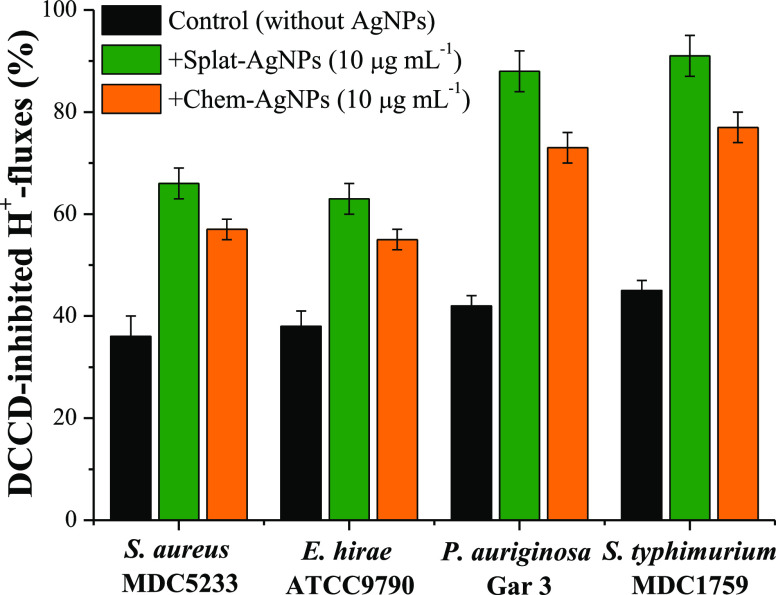
DCCD-inhibited energy-dependent H^+^-fluxes through the bacterial membrane in the presence of Chem- and Splat-AgNPs.

The energy-dependent H^+^-fluxes through the bacteria membrane were studied in order to determine the possible targets of Splat-AgNPs action. The energy-dependent H^+^-fluxes were suppressed by DCCD, an inhibitor of H^+^-translocating systems, in all bacteria by ∼40–45% (Figure 7). The addition of Splat-AgNPs led to a further decrease of DCCD-sensitive H^+^-fluxes in Gram-negative bacteria up to ∼90%; whereas Chem-AgNPs decreased DCCD-inhibited H^+^-fluxes in *P. aeruginosa* and *S. typhimurium* by ∼75%, respectively. Meanwhile, these fluxes in Gram-positive bacteria showed a lower susceptibility to both NPs (Figure 7). The interaction of Splat-AgNPs with membrane proteins, such as H^+^-translocating F_O_F_1_-ATPase, leads to alteration in membrane permeability and the ATP-associated metabolism of tested bacteria.^11,41^ Thus, ATPase inhibition and alterations in membrane potential cause inhibition of bacterial growth and cell death.

The general mechanism of the antibacterial activity of AgNPs is related to the release of free positively charged Ag^+^, their adsorption on the negatively charged surface of the bacterial membrane, interaction with membrane-bound proteins and their inactivation, and penetration into the cell, which leads to the disruption of the membrane structure and ion transfer across the membrane.^6,7,32^ The disruption of the structure of the bacterial membrane by AgNPs, as well as the formation of reactive oxygen species via the contribution of free Ag^+^, affects various metabolic processes of bacteria, resulting in the inhibition of growth and death of bacteria.^6,7^ Moreover, AgNPs increase the efficacy of traditional antibiotics against pathogenic bacteria.^59,60^

## 4 Conclusions

Our research has shown that *Spirulina*’*s* biomass can be a valuable low-cost platform for the biosynthesis of AgNPs. The eco-friendly and low-cost green synthesis of stable AgNPs with antimicrobial potential by an aqueous extract of *S. platensis* has been developed. The formation of Splat-AgNPs was confirmed by UV–vis spectroscopy. FTIR analysis revealed the involvement of biomolecular functional groups in the reduction of Ag^+^ to AgNPs. The various metabolites of *S. platensis* extract, such as proteins, flavonoids, organic acids, and alkaloids, can surround the Splat-AgNPs and stabilize them. The nanoscale range of Splat-AgNPs was identified by complementary DLS and NTA measurements and verified by TEM. Splat-AgNPs with a hydrodynamic radius size of ∼29 nm demonstrated high colloidal stability with a value of (−50) mV. Additionally, XRD and SAED confirmed the crystallinity of NPs, and EDS analysis indicated the presence of elemental Ag in large quantities in Splat-AgNPs. Antibacterial activity studies revealed that Splat-AgNPs exhibit pronounced bactericidal potential against selected Gram-positive and Gram-negative bacteria in comparison with Chem-AgNPs, in that Gram-negative bacteria (*P. aeruginosa* and *S. typhimurium*) demonstrated greater sensitivity to Splat-AgNPs compared to Gram-positive *E. hirae* and *S. aureus*. In that process, Gram-negative bacteria (*P. aeruginosa* and *S. typhimurium*) demonstrated greater sensitivity to Splat-AgNPs compared to Gram-positive *E. hirae* and *S. aureus*. Moreover, Splat-AgNPs significantly influence the DCCD-sensitive energy-dependent H^+^-fluxes in all bacteria, indicating membrane permeability changes and H^+^-translocating F_O_F_1_-ATPase activity changes. The obtained results contribute to an understanding of the antibacterial activity mechanism of NPs and provide a basis for the biotechnological production of *Spirulina*-mediated synthesis of stable AgNPs and their further applications in biomedicine.

## Data Availability

All data generated or analyzed during this study are included in this manuscript.

## Notes

Ethical approval: All human blood samples in this manuscript were obtained from healthy volunteers and used according to the “Human Rights and Biomedicine, Oviedo Convention” (CE, 1997) and approved by the National Center of Bioethics (Armenia).
